# Perspectives of users for a future interactive wearable system for upper extremity rehabilitation following stroke: a qualitative study

**DOI:** 10.1186/s12984-023-01197-6

**Published:** 2023-06-13

**Authors:** Chieh-ling Yang, Rochelle Chui, W. Ben Mortenson, Peyman Servati, Amir Servati, Arvin Tashakori, Janice J. Eng

**Affiliations:** 1 Department of Occupational Therapy and Graduate Institute of Behavioral Sciences, College of Medicine, Chang Gung University, Taoyuan City, Taiwan; 2 Department of Physical Medicine and Rehabilitation, Chang Gung Memorial Hospital, Chiayi, Taiwan; 3 Faculty of Applied Science and Faculty of Medicine, Undergraduate Program in Biomedical Engineering, University of British Columbia, Vancouver, Canada; 4 Centre for Aging SMART at Vancouver Coastal Health, Vancouver, BC Canada; 5 Department of Occupational Sciences and Occupational Therapy, University of British Columbia, Vancouver, Canada; 6 International Collaboration on Repair Discoveries, Vancouver, Canada; 7 Department of Electrical and Computer Engineering, University of British Columbia, Vancouver, Canada; 8 Department of Physical Therapy, University of British Columbia, Vancouver, Canada

**Keywords:** Stroke, Wearable technology, Rehabilitation, Upper limb, Qualitative study

## Abstract

**Background:**

Wearable sensor technology can facilitate diagnostics and monitoring of people with upper extremity (UE) paresis after stroke. The purpose of this study is to investigate the perspectives of clinicians, people living with stroke, and their caregivers on an interactive wearable system that detects UE movements and provides feedback.

**Methods:**

This qualitative study used semi-structured interviews relating to the perspectives of a future interactive wearable system including a wearable sensor to capture UE movement and a user interface to provide feedback as the means of data collection. Ten rehabilitation therapists, 9 people with stroke, and 2 caregivers participated in this study.

**Results:**

Four themes were identified (1) “Everyone is different” highlighted the need for addressing individual user’s rehabilitation goal and personal preference; (2) “The wearable system should identify UE and trunk movements” emphasized that in addition to arm, hand, and finger movements, detecting compensatory trunk movements during UE movements is also of interest; (3) “Both quality and amount of movements are necessary to measure” described the parameters related to how well and how much the user is using their affected UE that participants envisioned the system to monitor; (4) “Functional activities should be practiced by the users” outlined UE movements and activities that are of priority in designing the system.

**Conclusions:**

Narratives from clinicians, people with stroke, and their caregivers offer insight into the design of interactive wearable systems. Future studies examining the experience and acceptability of existing wearable systems from end-users are warranted to guide the adoption of this technology.

## Background

Stroke is a major cause of disability worldwide, with a global prevalence estimated at 101 million in 2019 [1]. Approximately 80% of cases experience upper extremity (UE) dysfunction, impacting the integration of the affected UE use into everyday life [2]. During the inpatient stage, individuals with stroke typically receive rehabilitation consisting of a baseline assessment, goal setting, intervention, and progress evaluation for their UE function [3]. After being discharged home, individuals with UE paresis still need further treatment such as outpatient and in-home rehabilitation to facilitate their UE recovery and maintain their function [4]. However, rehabilitation following stroke is often constrained by factors such as limited healthcare resources in hospitals and the community [5].

By tracking of physiological and body motion signals, wearable sensor technology may facilitate diagnostics and monitoring of people with UE paresis, which may lead to improved rehabilitation outcomes after stroke [6]. In stroke rehabilitation, repetitive-task training is one of the key principles to promote motor recovery [3]; i.e., more practice leads to better outcomes [7]. Interactive wearable systems can provide feedback about the affected UE via user interfaces to improve the user experience and motivation [6]. They are ideal for facilitating practice and could augment rehabilitation outcomes in clinical settings and in-home settings [8]. As wearable technology for UE rehabilitation is attracting increasing research interest, multiple review papers have been conducted to describe these technologies and summarize the applications [6, 8, 9]. These review papers have concluded that wearable sensor technology is helpful in UE rehabilitation post-stroke, while challenges regarding clinical implementation and uptake should be addressed to guide future research and development of the technology [6, 8, 9]. To promote clinical utilization of wearable sensor technology, the perspectives of users such as clinicians and people who have a lived experience with stroke are needed to inform the design and development of new technologies and to promote the adoption of currently available technologies [10].

Some studies have investigated the perceptions of users for technologies designed to help people with stroke recover movement of their upper limb [11–13]. One qualitative study exploring the perceptions of stroke patients on existing wearable robotic devices for upper extremity identified a need to develop new devices for UE rehabilitation [11]. A survey study by Hughes et al. suggested that improvements in the design of assistive technologies (e.g., easy to set up and use, fun, etc.) is essential for better and more effective upper limb stroke rehabilitation [12]. One qualitative study by our group examined the perceptions of clinicians regarding a specific prototype for detecting the number of grasp and release movements in individuals with stroke [13] and this led to the development of a “grasp counter” [14]. However, no studies have provided information on the design of an ideal wearable system that is designed for arm and hand rehabilitation for people with stroke. With our intent to develop a future wearable system for stroke UE rehabilitation, the current study aims to explore the perceptions of users (i.e., rehabilitation therapists, individuals with stroke, and caregivers of stroke patients) of a future interactive wearable system for stroke. We specifically explored what types of upper limb movements (including those of the arm, hand, and finger movements) and functional activities would be essential to capture.

## Methods

This study used qualitative methods to explore the perceptions of users (i.e., patients, their caregivers, and therapists) of a future interactive wearable system (a wearable device coupled with an interactive user interface) that measures upper extremity movements and provides feedback for individuals with stroke. The local university ethics boards approved ethical approval for this study (H20-02543). The COREQ (Consolidated criteria for reporting qualitative research) guidelines was used to report the qualitative data to ensure methodological quality and transparent reporting [15].

### Participants

A convenience sample of users (i.e., rehabilitation therapists, stroke patients, and their caregivers) was recruited. To be eligible to participate, participants had to be 19 years or older and had devices (a computer/laptop/tablet/phone with a webcam) and access to the Internet for videoconferencing. Physical and occupational therapists were recruited from a local rehabilitation hospital and private clinics. Therapists were eligible if they were currently working in poststroke UE rehabilitation. Individuals with stroke were recruited from former participants of studies who had provided consent to be contacted for study recruitment. Caregivers were recruited from the former participants’ primary caregivers. Individuals with stroke were eligible if they were (1) have had a stroke more than three months ago, (2) have the ability to pick up an object with the affected hand, and (3) able to understand, follow instructions, and communicate verbally. All individuals interested in participating met the inclusion criteria and provided signed informed consent were included.

### Data collection

Individual semi-structured interviews were conducted via videoconferencing using Zoom software (Zoom Video Communications Inc) in the interviewee’s own home or an alternative private venue according to their preferences. The Zoom sessions were audio and video recorded. Participants could turn off their video in the Zoom sessions if they did not want to be video recorded. All interviews lasted approximately 60 min and were conducted by the lead author, a trained researcher and occupational therapist having prior experience in administering qualitative research. An honorarium was offered to all participants for their time. Separate interview guides were developed for participants with stroke, their caregivers, and therapists. The lead author developed the guides reviewed by an interdisciplinary team consisting of three physiotherapists (JJE, TK), two occupational therapists (CY and LS), one physician (JY), two engineers (PS and AS), and one qualitative research expert who is also an occupational therapist (WBM). All interview guides consisted of open-ended questions and more specific probing questions to explore the participant’s perspectives of the following topics: (1) perspectives about using an interactive wearable system to detect UE motion coupled with an interactive user interface to provide feedback; (2) essential features for an ideal future wearable system to detect UE movements and activities; (3) opinions about a specific prototype coupled with an interactive user interface that can detect wrist, hand, and fingers and provide feedback about the affected UE; and (4) a list of movements or activities that they would prioritize to be included in the device for use. Participants were informed that the aim of the interview was to obtain their opinions on the development of a future wearable device coupled with interactive user application software for stroke UE rehabilitation. The specific prototype was described as “a sensor that can detect force and movements of the hand, finger, and wrist movements with interactive user application software which can motivate users and provide feedback.” During an interview, the interviewer restated or summarized information provided by the participant and then asked the participant if this summary was accurate. All interviews were audio-recorded, transcribed verbatim, and reviewed for accuracy.

Basic demographic information was also collected for all participants. For therapists, sex, age, professional background, work setting, and years of practice were collected. For participants with stroke, age, time since stroke, sex, side of paresis, and dominant hand were collected. For caregivers, sex, age, and care recipient’s time since stroke were collected.

### Data analysis

All interview transcripts were analyzed using conventional content analysis where coding categories are derived directly from the text data. No specific framework was used for analysis as existing research literature on this topic of interest is limited [16] and as the aim of the study was to inform the development of a future wearable system. To ensure the trustworthiness, we used the following strategies. First, the interviewers and data analysts wrote field notes and analytic notes to record their thoughts and emerging ideas when interviewing participants and approaching the data. This process was used in self-reflection when reviewing the findings. Second, the two authors (CY and RC) read and re-read the transcripts prior to coding the data to ensure familiarization with the data. Statements were inductively coded to initial codes that encompass the statement’s meaning by CY and RC independently. They then worked collaboratively to consolidate and group the initial codes into broad categories. Through an iterative process, themes, subthemes, and interpretations were drafted based on the coding. To reach a consensus, these themes and subthemes were refined and discussed with two other authors (JJE and WBM). In addition to the themes and subthemes developed from the data, design considerations for the feedback features of an ideal interactive wearable system were also extracted from the data. Data collection ended when data saturation was achieved as agreed upon by two authors who analyzed the data (CY and RC). Microsoft Excel was used for the analyses.

## Results

A total of 21 interviews (9 patients, 2 caregivers, and 10 therapists) were carried out over October 2020 and January 2021. Table 1 outlines the demographic characteristics of each participant, using the identifier “S,” “C,” and “T” to denote participants with stroke, caregivers, and therapists. Four main themes with subthemes were extracted from the data (Table 2): (1) Everyone is different; (2) The wearable system should identify UE and trunk movements; (3) Both quality and amount of movements are necessary to measure; (4) Functional activities should be practiced by the users. Table 2 provides illustrative quotes for each subtheme.

**Table 1:** Participant characteristics

Participant no.	Sex	Dominant hand	Side of paresis	Age range, years	Time since stroke, months
S1	M	R	L	50–54	23
S2	F	R	R	50–54	22
S3	F	R	L	55–59	123
S4	M	R	L	60–64	36
S5	F	R	L	50–54	69
S6	M	R	L	70–74	154
S7	M	R	L	65–69	62
S8	M	R	L	65–69	36
S9	M	R	R	70–74	56

Participant no.	Sex	Age range, years	Care recipient’s time since stroke, months
C1	F	60–64	25.7
C2	F	50–54	115.4

Participant no.	Sex	Age range, years	Professional background	Work setting	Clinical experience, years
T1	F	45–49	PT	Private clinic, research lab	23
T2	F	30–34	PT	Private clinic, research lab	4
T3	M	30–34	PT	Private clinic, research lab	9
T4	F	30–34	OT	Rehab centre	2
T5	F	25–29	OT	Rehab centre	1.5
T6	F	35–39	PT	Private clinic	10
T7	F	40–44	PT	Rehab centre	7
T8	F	40–44	PT	Rehab centre	7
T9	F	35–39	OT	Rehab centre	7
T10	F	40–44	OT	Rehab centre	13

**Table 2:** Themes and subthemes

Themes	Subthemes
1. Everyone is different	(1) Allow for personalization and customization
	(2) Consider different roles for the dominant and non-dominant hands
2. The wearable system should identify UE and trunk movements	(1) UE Impairment-based movements
	(2) UE Functional movements
	(3) Trunk movements during UE movements
3. Both quality and amount of movements are necessary to measure	(1) Quality of movements
	(2) Amount of movements
	(3) Quality and amount of movements may share some common purposes
	(4) Quality and amount of movements may serve different purposes
4. Functional activities should be practiced by the users	(1) Functional movements
	(2) Everyday activities
	(3) Principles of progression

### Theme 1: everyone is different

This theme focused on the fact that a wearable device coupled with an interactive user interface should have the ability to address an individual’s specific needs for UE rehabilitation as every stroke patient is different in terms of their impairment levels, rehabilitation goals, and personal preferences for devices and user interfaces. For instance, one participant with stroke (S7) stated: “I would think the device should be tailored to the patient. What I mean is the time after they had a stroke. Secondly, what kind of differences or challenges do they have after their stroke. It has to be designed for that type of patient.” There were two subthemes: (1) Allow for personalization and customization; and (2) Consider different roles for the dominant and non-dominant hands.

In subtheme 1, when asked about the features that an ideal interactive wearable system might possess, participants expressed that it should allow users to personalize and customize to meet the needs of any given individual. For example, the functionality to select the joints/segments measured (e.g., fingers, wrist, forearm), parameters measured (e.g., speed, joint angles), and types (e.g., encouraging words, smiley faces) and amount of feedback provided. Participants described that UE movements and parameters that a device captured should be aligned with each individual’s level of impairment and rehabilitation goal. For instance, one therapist (T3) mentioned: “Thinking about the range of impairment you see some individuals have. You know, you might only care about their shoulder because they have no ability in their hand, whereas for the high functioning people, I would want to know what someone’s every joint was doing.”

Regarding the types and amount of feedback provided by the device, participants highlighted the need for customization according to personal preference for feedback. For instance, one therapist (T2) stated: “Some clients really like to see a lot of data, and some don't.” In addition, feedback in a linguistic form may not be helpful for individuals with aphasia. A list of ideal feedback features for a future wearable device coupled with an interactive user interface was summarized in Table 3.

**Table 3:** Summary of ideal feedback features for a future wearable device coupled with an interactive user interface

Feedback features	Examples
Showing achievement/progress	– Showing how many days the user has completed goals – Comparison to the day before/ prior session – Showing if the user completes the daily/weekly goal (e.g., completion ring, bar) – Showing percentage of improvement compared to the nonaffected side
Rewards	– Smiley faces/starts/coins/badges – Encouraging words (e.g., nice job, way to go) – Point system – Leaderboard: the user’s ranking compared to other users
Feedback on desired/undesired movement patterns	– Video demonstrations on desired/undesired movement patterns – Reminders while performing undesired movement patterns
Formats	– Charts/ graphs – Numerical data (e.g., scores, grades)

A few therapists also indicated that individuals with dominant side versus non-dominant side affected might have different needs for functional recovery. For individuals with dominant side affected, the goal may be to enhance the active role of the dominant UE (e.g., grasping, object manipulation), while for individuals with non-dominant side affected, the goal may be to improve the use of the non-dominant UE as a supportive role. For instance, one therapist (T1) stated: “The arm is used in so many movements, so it totally depends on whether or not it's the non-dominant or dominant hand, to begin with as well. If they're right-handed, and their weakness is on their left hand, I would like to see that arm being used more as a supportive arm.”

### Theme 2: the wearable system should identify UE and trunk movements

In this theme, participants described various UE segments, functional UE movements, and trunk movements during UE movements that an ideal wearable system should capture. There were 3 subthemes: (1) UE impairment-based movements; (2) UE functional movements, and (3) Trunk movements during UE movements. For instance, one therapist (T6) said, “Ideally, I want to capture it all (arm, hand, and fingers) and even trunk to some degree because some people are moving their arm, but they’re also moving their trunk with it.”

In subtheme 1, participants expressed that various impairment-based UE movements should be detected, such as shoulder flexion, elbow extension, wrist extension and flexion, forearm supination and pronation, finger opposition, and individual finger movement. One S4 stated: “It’ll be a good idea to measure how you can move each finger individually.” and as one T10 stated: “……having some capacity to capture the wrist activity or the position of the wrist would be really useful because sometimes you can get really like gross finger flexion that isn't necessarily purposeful.” In addition, some therapists stated that any compensatory movements that occurred in the shoulder and elbow should also be identified.

In subtheme 2, participants highlighted the need for capturing functional UE movements such as reaching to different heights and directions, grasping and releasing objects with various shapes and sizes, in-hand manipulation, writing, texting, and assisting with ambulation (e.g., using a walking stick, arms swinging while walking). For instance, one T2 stated, “……like reaching at different heights. And like the extent of reach compared to feel like if they're just reaching close to them versus reaching far.”

In subtheme 3, participants, especially therapists, identified that compensatory movements occurred in the trunk (e.g., trunk rotation, anterior trunk displacement), during UE movements should be captured. As T8 stated, “If you're looking at arm movement, you need to measure what the trunk is doing. So that you have a relative movement of the wrist with respect or of the rest of the arm with respect to the trunk.”

### Theme 3: both quality and amount of movements are necessary to measure

This theme reflected that how and why participants would use a wearable system to measure how well (quality) and how much (amount) of UE movements. Participants emphasized that both quality and amount of movements should be measured in and outside of therapy. As T1 stated, “[talking about quality and amount of movements] …… I would make sure I have a video on where I'm going for quantity to make sure that it's being done in as close to a normal movement as possible.” There were 4 subthemes: (1) Quality of movements; (2) Amount of movements; (3) Quality and amount of movements may share some common purposes; (4) Quality and amount of movements may serve different purposes.

In subtheme 1, participants indicated that the quality of movements could be informed by biomechanical information such as force, coordination, speed, range of motion, accuracy, smoothness, and muscle activation patterns. For instance, S6 stated, “If I could go back to reading the newspaper with both hands, that would be perfect, but it's not so much holding the newspaper. It's, it's holding it without crushing it.” In addition, some therapists expressed that an ideal device should have the ability to distinguish between desired and compensatory movement patterns.

In subtheme 2, participants emphasized that the duration of use and repetitions of the affected UE would be ideal parameters to quantify the amount of UE activity. For instance, C1 stated, “I feel like it would be a good incentive to make targets. …… And it can be used as a reminder to do certain things.”

In subtheme 3 and 4, participants discussed the purposes of using the device for training inside and outside of therapy sessions. Subtheme 3 describes the common purposes of quality and amount of movement that the participants would like the device to measure. Participants envisioned that the device would be used to track changes and progress to indicate the user's motor recovery and show the effectiveness of given rehabilitation therapy. They imagined the interactive user interface which is coupled with the device would provide instructional feedback on the affected UE for the users to promote desirable movement patterns, avoid compensatory (undesirable) behaviors, augment UE training adherence, increase motivation and self-management, and set tailored goals for exercises. Both quality and amount of movements should be captured in supervised clinical settings and in-home and community settings to understand how the users utilize the affected UE during the day, how they carry over from clinic to actual use in home and community, and to monitor adherence and compliance to a home program. For instance, T2 expressed, “Ideally, I would like both (in therapy and outside therapy). The way I always look at my treatments is like, they can perform really well while they're doing the specific exercise (in therapy), but how is it translating to their actual function throughout their day (outside therapy)”.

Subtheme 4 described that quality and amount of movements might be used for different purposes. Some therapists argued that using indicators for the quality and amount of movement should depend on the treatment goal. For clients performing incorrect movements patterns, the quality of movement should be of priority. In contrast, the amount of movements should then be emphasized for clients able to execute movements with desirable quality. Some therapists mentioned that the quality of movement is more useful outside of therapy sessions as it would capture compensatory movements and make sure users perform daily tasks and exercises properly without therapist supervision. Some therapists pointed out that the quality of movements is more suitable for higher-level clients while the amount of movements is more ideal for lower-level clients. Some therapists emphasized that lower-level patients should focus on increasing the use of their affected UE. Once they use their affected hand more, quality should be added to the treatment goal to prevent injury and ensure appropriate movement patterns. Some therapists doubted if providing feedback regarding the quality of movements was necessary during therapy sessions unless the parameters could not be seen visually by therapists as described by T3: “I would see a device being an assessment piece to do something that I can’t see on my own, with my own eyes. Whether it was telling me muscle activity or it was able to quantify better someone’s movement pattern than I can do visually.” In contrast, some therapists foresaw a device for capturing the quality of movements such as speed or angles that they may not be able to quantify with their eyes. Some therapist participants envisioned using a device for collecting information about the amount of movements in therapy sessions. Although therapists may possess different views on how to use the information related to the quality and amount of movements, therapists generally expected an ideal wearable system to complement the assessment of UE movements done by the therapist in therapy sessions and to extend the evaluation of UE use patterns beyond supervised clinical settings into home and community settings.

### Theme 4: functional activities should be practiced by the users

The final theme centered around the movements and tasks related to everyday activities that are of priority for users to practice and the principles of progression for these movements and tasks. There were 3 subthemes: (1) Functional movements; (2) Everyday activities; (3) Principles of progression.

In the first subtheme, participants described specific functional movements that the potential users should focus on. Movements included reaching various heights and directions and grasping and releasing objects of different sizes and shapes. As mentioned by S6: “I guess when I think, I think of those little kid blocks, so it’s a bit smaller, so you’re getting a bit more of a lateral pinch grasp, right, versus a (blob) picking up a full grasp.” In the second subtheme, participants felt that activities of daily living (e.g., feeding, grooming, dressing), instrumented activities of daily living (e.g., cooking, doing laundry, writing, texting, typing, driving), and leisure activities (e.g., playing cards, music instruments, sports) are of interest to be in the design. As T4 stated: “I would love to see the ability for different things related to activities of daily living practice, so do buttons, or tie shoelaces.” and as S1 stated, “For me, it’s doing the laundry itself is quite good.” In the third theme, participants illustrated the general principles for exercise progression to inform the device’s design coupled with an interactive user interface. Two principles were described: (1) From single to multiple joints/planes/components; and (2) Gradually increase speed, range, force, or variability of the movements or tasks. As described by S5, “From drinking of the cup, it goes from being able to reach to the cup and opening the glass to holding the cup to bringing the hand back. So it's all the movements that a normal person can do, that's broken down into individual steps.” and by S2, “Distance from very small to them gradually more distance. And going in different directions, going from left to right, laterally, speediness.”

In addition to the above main themes and subthemes, the data also revealed many essential design considerations for the feedback features of an ideal interactive wearable system that are presented in Table 3. Four feedback features with examples were extracted from the data: (1) Showing achievement/ progress; (2) Rewards; (3) Feedback on desired/undesired movement patterns; (4) Formats.

## Discussion

This study investigated users’ (i.e., rehabilitation therapists, individuals with stroke, their caregivers) attitudes toward a future interactive wearable system for capturing UE movements and activities poststroke. The interview data has informed important considerations of a wearable system that tracks explicitly arm, hand, and finger movements and the coupled interactive user interface that provide feedback about movements. Moreover, it has also informed the development of similar wearable technologies.

### The need to include diverse functional UE movements

Aligned with previous review studies on wearable technology [8, 17, 18], therapists, participants with stroke, and their caregivers emphasized the importance of personalization and customization of an ideal future wearable system to increase usability. Marked heterogeneity in clinical presentation and recovery patterns among individuals with stroke were the core reasons for the necessity of individualization of all participants. Among the myriad of available rehabilitation treatment options, it is still challenging to decide the most optimal intervention [19]. In addition, a client’s personal preferences for movements of interest and feedback types were brought out consistently, which is in line with our previous qualitative paper [13]. A few therapists highlighted the differential roles of dominant and non-dominant hands in real-life activities that might influence how therapists set tailored goals for clients. This is also supported by literature indicating differences in patterns between the dominant and non-dominant hand affected [20, 21]. For example, therapists might focus on improving the dexterity of the dominant affected hand while emphasizing the supportive role (i.e., use the arm for support) of the nondominant affected hand. Nevertheless, it is still essential to enhance the motor recovery of the affected UE as most activities in daily living require the use of both sides together. Better functional performance in activities in daily living is positively associated with the help of both arms together following stroke [21]. Thus, there is a need to include various functional UE movements in a wearable system to make these systems more applicable to a wide range of stroke patients.

### The need to capture the amount and quality of UE movements

It is not surprising that rehabilitation therapists wanted to know how much and how well clients used their affected UE. Aligned with a growing emphasis in rehabilitation literature, therapists also expressed the need for capturing the amount of movement to monitor the therapy dose and actual use of affected UE outside of therapy sessions [9, 22]. Consistent with our previous qualitative paper [13], the ability of a future wearable system to capture the quality of UE movements was salient among participants. This is also aligned with our findings that the therapists want to detect trunk movements during UE movements to differentiate compensatory and normal movement patterns in Theme 2 (The device should capture UE and trunk movements). Moreover, the benefits to reduced compensatory trunk movements and the importance of distinguishing motor restitution versus compensation by using kinematic and kinetic measurements have also been suggested in the current American Heart Association/American Stroke Association guideline [23] and the recommendations from the Stroke Recovery and Rehabilitation Roundtable [24].

Although an ideal device that could capture how much and how well the user is using their affected UE was highlighted by participants, therapists had different views on the priority of which parameter should be focused. For example, some therapists pointed out that increasing the amount of UE movements should be prioritized for lower-level clients. In contrasts, some therapists argued that the quality of movements should be addressed first to ensure proper forms and prevent injuries especially when therapy supervision is not available. The different perspectives on when and how to use these indicators may result from variations in a therapist’s clinical judgment and reasoning, which may be affected by work settings, years of clinical experience, and a client’s recovery stages and functional levels [25]. Further studies could explore the influence of these factors on therapists' perspectives on the use of wearable technology. Despite various perspectives, we suggest that a wearable system should include the functionality to quantify both the amount and quality of UE movements for the users to decide when, how, and what indicator(s) to be used.

### Movements and tasks related to everyday activities are of interest.

Aligned with exiting knowledge of contemporary task-oriented rehabilitation interventions, participants highlighted the desire for users to practice goal-directed movements of functional tasks instead of impairment-based movements [26, 27]. Participants with stroke and their caregivers provided more specific examples of everyday activities related to their and their care receiver’s own personal attributes. At the same time, therapists tended to describe what they usually have their clients practice. Both therapists and participants with stroke were able to provide insights on how they would progress the movements and tasks of interest, which is consistent with one of the principles of motor learning, increasing the level of difficulty and providing optimal challenges [28]. Thus, we suggest that an ideal interactive wearable system for stroke UE rehabilitation should provide practice of functional and goal-directed tasks. Impairment-based movements (e.g., isolated wrist flexion and extension, hand opening) that assist in performing everyday activities should also be incorporated within the spectrum of a device. Furthermore, exercise activities embedded in the wearable system should always be adjustable to provide the “just-right challenge.”

### Ideal design of a future interactive wearable system for stroke UE rehabilitation

Based on the perspectives of our participants, we envision that the ideal design of a future interactive wearable system for UE stroke would capture all involved segments during UE movements, including shoulder, arm, wrist, fingers, and trunk. Functional movements that are relevant to everyday activities would be practiced with the device. Ideally, the interactive user interface should have features to provide feedback on the quality and quantity of the movements. The whole system (i.e., the device and the interface) should have the ability to personalize for different needs of the users (e.g., increase or decrease difficulty, have a selection of movement for practice). Although the literature has shown that robotic systems may have the capability to contain all the above features [28], the accessibility of such features for general users is limited. Therefore, we envision that a light and portable device on the hand, such as a glove or ring on the hand, coupled with sensors on the shoulder and elbow, would meet the requirements of the users and be feasible for design. A portable camera-based system to recognize movements might be an alternative, but metrics such a as grip force would be difficult to measure. The device would be able to recognize the quality and quantity of functional UE movements (e.g., reaching and grasping an everyday object). The interactive user interface shown on a laptop or tablet would offer enjoyable games and motivational feedback paired with movements being practiced. Users would be able to view the record of the treatment progress and adherence on the interface.

### Limitations and future research directions

There were three main limitations to this study. First, participants did not see a prototype of the future wearable device coupled with an interactive user interface. While it may be hard for participants to imagine the future wearable system hypothetically, providing a tangible thing can limit creativity. Second, the sample of therapist participants lacks male therapists but represents our local setting where there are primarily female therapists working in stroke rehabilitation. Lastly, all participants except one caregiver were from an urban area (the Greater Vancouver area), limiting the transferability of the findings to other contexts. As this study aimed to explore users’ views towards a future wearable system that include a wearable sensor to detect UE movements and a coupled user interface to provide feedback about the movements, future studies examining the experience and acceptability of existing wearable systems are thus warranted to guide the adoption of this technology.

## Conclusions

Therapists, individuals with stroke, and their caregivers reported essential considerations for designing and developing an interactive wearable system for UE rehabilitation. An ideal interactive wearable system should have the ability to be customized to address an individual’s needs, capture both the amount and manner of UE movements, and incorporate functional and goal-directive movements with multiple levels of difficulty for users to practice.

## Data Availability

Interview transcripts and audio files are not publicly available due to the risk of identifying the study participants.

## Abbreviations

UE: Upper extremity
COREQ: Consolidated criteria for reporting qualitative research
